# Disruption of *INOS*, a Gene Encoding *myo*-Inositol Phosphate Synthase, Causes Male Sterility in *Drosophila melanogaster*

**DOI:** 10.1534/g3.118.200403

**Published:** 2018-07-10

**Authors:** Natasha A. M. Jackson, Angelina M. Flores, Elizabeth D. Eldon, Lisa S. Klig

**Affiliations:** * Department of Biological Sciences, California State University Long Beach, Long Beach, California

**Keywords:** development, fertility, inositol, metabolism, reproduction

## Abstract

Inositol is a precursor for the phospholipid membrane component phosphatidylinositol (PI), involved in signal transduction pathways, endoplasmic reticulum stress, and osmoregulation. Alterations of inositol metabolism have been implicated in human reproductive issues, the therapeutic effects of drugs used to treat epilepsy and bipolar disorder, spinal cord defects, and diseases including diabetes and Alzheimer’s. The sole known inositol synthetic enzyme is *myo*-inositol synthase (MIPS), and the homolog in *Drosophilia melanogaster* is encoded by the *Inos* gene. Three identical deletion strains (*inos^ΔDF^*/CyO) were constructed, confirmed by PCR and sequencing, and homozygotes *(inos^ΔDF^*/*inos^ΔDF^*) were shown to lack the transcript encoding the MIPS enzyme. Without inositol, homozygous *inos^ΔDF^* deletion fertilized eggs develop only to the first-instar larval stage. When transferred as pupae to food without inositol, however, *inos^ΔDF^* homozygotes die significantly sooner than wild-type flies. Even with dietary inositol the homozygous *inos^ΔDF^* males are sterile. An *inos* allele, with a *P*-element inserted into the first intron, fails to complement this male sterile phenotype. An additional copy of the *Inos* gene inserted into another chromosome rescues all the phenotypes. These genetic and phenotypic analyses establish *D. melanogaster* as an excellent model organism in which to examine the role of inositol synthesis in development and reproduction.

The metabolism of *myo*-inositol plays a role in fertilization and early embryonic development, and alterations have been implicated in reproductive issues, cancer, neurodegenerative disorders, spinal cord defects, epilepsy, and bipolar disorder (Croze and Soulage 2013; Bevilacqua *et al.* 2015; Koguchi *et al.* 2016, Ye and Greenberg 2015; Yu *et al.* 2017). *Myo*-inositol is a six-carbon sugar alcohol found in eukaryotic and many prokaryotic cells. It is the precursor for the phospholipid membrane component phosphatidylinositol (PI), and has important roles in signal transduction, endoplasmic reticulum stress, and osmoregulation (Henry *et al.* 2014; Chow *et al.* 2015).

There are three ways an organism can acquire inositol. The first is through inositol transport from the extracellular environment (Spector and Lorenzo 1975). The second is via recycling by dephosphorylation of inositol phosphates (Berridge and Irvine 1984). The third is synthesis via a two-step process from glucose-6-phosphate, for which the first step is catalyzed by *myo*-inositol-3-phosphate synthase (MIPS) (Loewus and Kelly 1962; Eisenberg *et al.* 1964). The properties and catalytic mechanisms of MIPS are similar in animals, plants and yeast (Chen and Charalampous 1964; Loewus *et al.* 1983; Klig *et al.* 1994; Molina *et al.* 1999). Currently the genomes of more than one hundred organisms, ranging from microbes to man, contain annotated orthologs which encode MIPS (NCBI). In most organisms it is a homo-tetramer (approximately 62 kD per subunit) (Henry *et al.* 2014).

High levels (millimolar concentrations) of *myo*-inositol have been detected in seminiferous tubule fluid of several mammalian species. To establish and maintain this high level, one hundred times more than in plasma, there must be some combination of extremely high levels of synthesis, transport against a large concentration gradient, and a barrier to loss of inositol (cell-cell contact and unidirectional active transport) (Lewin *et al.* 1976). In mice higher levels of MIPS mRNA have been demonstrated in the Sertoli cells, pachytene spermatocytes, and the round spermatids of the testis (Chauvin and Griswold 2004). The testicular barrier isolates these cells from exogenous molecules (Lefever *et al.* 2015, Mruk and Cheng 2015). Similarly, the somatic cyst cells form the barrier in *D. melanogaster* that tightly encapsulates developing spermatids in later stages of spermatogenesis, preventing transport of extracellular compounds (Fairchild *et al.* 2015).

*D. melanogaster* development has been extensively studied. After embryogenesis first-instar larvae emerge and feed nearly continuously for one day, then molt, becoming larger second-instar larvae, which continue to feed for another day. After molting again, they become third-instar larvae, which feed for three days before pupating. Pupal development proceeds for three to five days after which the adults eclose. The typical lifespan of an adult is 20-60 days. The *D. melanogaster* ovary begins forming during embryogenesis, and the ovary structure develops during pupal stages. In the adult ovary there are typically sixteen to twenty ovarioles. Fourteen identifiable stages of oocyte development are evident in linear order in ovarioles. Upon eclosion, the most mature oocytes of *D. melanogaster* females are in stage seven (previtellogenic). Approximately one and a half days later the ovarioles of these females will have all fourteen stages present, including mature eggs (Spradling 1993; McLaughlin and Bratu 2015). Male spermatogenesis also begins during embryogenesis. Testes of third-instar larvae are ovoid containing pre-meiotic stage germ-line cells. In pupae, testes elongate, coil, and the first spermatids may also begin to appear. In the adult male, the germline stem cells are located at the apical end of the testis. These cells undergo mitotic division, producing a stem cell and a primary spermatogonial cell, which becomes enclosed in two somatically derived cyst cells. Four mitotic divisions yield sixteen spermatocytes, these then undergo meiosis producing 64 haploid spermatids. Later steps of spermatid maturation include nuclear shaping and individualization. Actin has a major role in the individualization process. Coiling occurs, the mature sperm are released into the testis lumen, and then transferred to the seminal vesicle for storage until they are used for fertilization (Fuller 1993, White-Cooper 2009).

High throughput expression analyses reveal that *Inos*, the gene encoding *D. melanogaster myo*-inositol-3-phosphate (MIPS), is highly expressed in testes and the head (Chen *et al.* 2014). The *D. melanogaster Inos* gene is located on chromosome 2 at band 43C3. A *D. melanogaster Inos* cDNA was cloned and expressed yielding a 565 amino acid MIPS protein with a molecular weight of 62.3 kD (Park and Kim 2004).

In the current study, *myo*-inositol synthesis and its role in growth, development, and reproduction, were explored in the model organism *D. melanogaster*. A precise deletion of the sole inositol biosynthetic gene, *Inos*, was generated. MIPS mutants have been reported in plants (Donahue *et al.* 2010) and many unicellular organisms (Klig *et al.* 1994; Molina *et al.* 1999); this may be the first report examining the phenotype of a MIPS gene deletion in animals. Homozygous *inos^ΔDF^* deletion embryos hatch, but without dietary inositol, they die within a few hours as first-instar larvae. If pupae grown with inositol are transferred to food without inositol, homozygous *inos^ΔDF^* deletion adults have a significantly shorter lifespan than wild-type flies. Surprisingly, even on rich food with dietary inositol the deletion homozygotes (*inos^ΔDF^*) the males are sterile. No sperm were observed in their seminal vesicles. The physiological basis of this sterility was further examined. A rescue construct containing an additional copy of the *Inos* gene on another chromosome restores the growth, development, and viability of homozygous deletion larvae and adults on food without inositol. The male sterile phenotype was also complemented by this construct. These studies contribute to the understanding of the role of inositol synthesis in growth, development, and reproduction.

## Materials and Methods

### Fly stocks and maintenance

Flies were maintained in standard laboratory conditions at 18° or 25° and 70–80% humidity on a 12hr: 12hr light-dark cycle. Stocks from the Bloomington Drosophila Stock Center include Canton-S (#1, CS), Oregon R (#5, OR), P{hsFLP}*y^1^w*^-^; *sna*^Sco^ /CyO (#1929), *y^1^w*^-^; P{SUPor-P} *Inos* ^KG07679^/CyO, S*bw^1^; *ry*^506^ (#14921, hereafter identified as P-*inos^KG07679^*), *w*^-^ ; *sna*^Sco^ / CyO, S*bw^1^ (#3198), and *w*^-^ ; *sna*^Sco^ / CyO, S*bw^1^ P{*Act*GFP *w*^-^ }CC2 (#9325, hereafter identified as CyO^GFP^). Stocks from Exelixis Harvard Medical School include w^-^ ; P{XP}d00881 (#D00881, hereafter identified as D{XP+}) and w^-^ ; P{WH}f00895 (#F00895, hereafter identified as F{WH-}).

All fly stocks were grown on either rich food (https://bdsc.indiana.edu/information/recipes/bloomfood.html) or chemically defined food. Inositol-free chemically defined food was prepared by merging the protocols of Sang (1955), Falk and Nash (1974), and Grandison *et al.* (2009). Vitamin mix and a stock solution of amino acids were used (Grandison *et al.* 2009) as follows. Various concentrations of inositol (0, 0.5mM, 170mM) were added as indicated in the text and figures. One hundred milliliters (mls) of defined food were prepared by dissolving 0.613g vitamin mix in 45mL of autoclaved agar solution (3g/100ml), adding 10mL of 10x amino acids stock, choline to 1mM and sucrose to 0.2M. 300μL of 30% Tegosept was added to the 100mL of food.

### Fertility and Development Tests

Fertility tests were performed with 2-5 day old flies, transferred to the food indicated and allowed to mate for twenty-four hours. GFP-marked CyO balancers were used to distinguish heterozygotes (*inos^ΔDF^*/ CyO^GFP^) from deletion homozygotes *(inos^ΔDF^*/ *inos^ΔDF^*, GFP-negative) throughout development. The number of eggs laid within the next twenty-four hours, and the number of eggs hatched within subsequent forty-eight hours, were recorded. Progeny were monitored until adulthood. Larval stages were determined using mouthhook morphology. GFP was visualized using a Nikon SMZ1500 dissecting microscope with epifluorescence using a GFP-B filter cube. A fixed number of males and females (4:6) were maintained in each vial for the survivorship experiment.

### Generation of *Inos* Gene Deletion

MIPS deletion flies were generated through the use of the FLP-FRT system. Fly strains with FRT containing inserts that flank the MIPS gene were purchased from the Exelixis Collection at the Harvard Medical School. The deletion strains (*inos^ΔDF^*) were generated by excising the DNA between the elements (Parks *et al.* 2004). The genetic selection of the deletion strains is described in the results section.

### Creating a Rescue Strain With a Second Copy of the *Inos* Gene

A genomic BAC clone (CH322-61014) CHORI (Children’s Hospital Oakland Research Institute), harboring a section of chromosome 2 from 596 base pairs upstream of the *Inos* gene to 13,275 base pairs downstream (P[acman] Resources Genome Browser, http://flypush.imgen.bcm.tmc.edu/cgi-bin/gb2/gbrowse/getbac/; Venken *et al.* 2009), was microinjected and inserted at an attP site on chromosome 3 (Genetics Services Inc.). A homozygous rescue strain (w^-^; *inos^ΔDF^*/ *inos^ΔDF^*; res/res) was created by crossing the *Inos* rescue chromosome into the *inos^ΔDF^* deletion strain.

### PCR

DNA was extracted (Huang *et al.* 2009) and PCR amplified using primers for the WH- (CCTCGATATACAGACCGATAAAAC) and XP+ (TACTATTCCTTTCACTCGCACTTATTG) elements (Parks *et al.* 2004). Primers were designed for the *Inos* upstream flanking genomic DNA (GAGCTAGTGGGAAATGCAAGG) and downstream genomic DNA (ATTCGGTTAGTTCCCGCCAG). The bands were excised using QIAquick Gel Extraction Kit (Qiagen, Germantown, MD), and sequenced (Eurofins Operon).

### RT-PCR

Total RNA was extracted from 2-5 day old flies grown on rich food using Trizol (Life Technologies) (Green 2012). Total RNA (5µg) was DNase treated using the DNA-free Kit (Ambion) and then reverse transcribed with Moloney Murine Leukemia Virus Reverse Transcriptase (M-MLV RT) (Promega), and in other experiments with Avian Myeloblastosis Virus Reverse Transcriptase (AMV-RT) (Promega). The following primers were used: forward *Inos* 5′ UTR TTCCAGAAGCAAGCACATTG and reverse *Inos* 3′ UTR TTTCGTAGTTTATCCAACTAAAACCA, forward *Rp49* GGATCCAGCTTCAAGATGACCATCCG and reverse *Rp49* CCAGGAACTTCTTGAATCCG. Size standards used were 1kb (Promega) or 100bp (Fisher). RT-PCR products were visualized with agarose gels (0.5–2%). The bands were excised using QIAquick Gel Extraction Kit (Qiagen, Germantown, MD), and sequenced (Eurofins Operon).

### *Drosophila melanogaster* ovary and seminal receptacle dissection and mounting

All of the females were six to nine days old when dissected. Non-virgins were allowed to mate from eclosion for nine days. Ovaries and seminal receptacles were dissected and washed in Ringer’s Solution (128mM NaCl, 5mM KCl, and 2mM CaCl_2_). Seminal receptacles were examined, using differential interference contrast microscopy with a 40X objective, for 2-4 females per strain or condition. Approximately twenty ovarioles per ovary were examined from 3-8 females per strain or condition. Ovaries and seminal receptacles were viewed on a Nikon E600 microscope and images were captured using a Retiga 2000R camera (Q imaging). Entire ovaries were scanned and the number of egg chambers at each stage was tabulated (Spradling 1993).

### *Drosophila melanogaster* Testes Dissections, Mounting, and Staining

For phase contrast imaging, testes from a minimum of five males per strain were dissected in Hoyle’s medium (Baccetti *et al.*, 1979), mounted in 1X PBS, and viewed using an EVOS Fl Auto Imaging System. For confocal microscopy, testes were dissected, fixed with 4% formaldehyde in 1X PBS, and stained with rhodamine phalloidin (Cytoskeleton, Inc.) at 1:1000 in 1X PBS (Bonaccorsi *et al.* 2011). At least thirteen males per strain were dissected, typically both testes were examined. The testes were placed in depression slides with approximately 20µl of 2µg/ml DAPI in 1X PBS. Samples were viewed using an Olympus FV 1000 fluoview confocal laser scanning biological microscope.

### Data Availability

Strains available upon request. All the relevant data are within the paper.

### Statistical analyses

Standard error was calculated for all experiments. The *p*-values were determined using student’s *t*-test for the data in figures 3 and 4, the Mantel-Cox log-rank test was used to analyze the data in figure 5, and chi-square analyses were used to analyze the data in figure 6.

## Results and Discussion

High levels of inositol synthesis, via *myo*-inositol-3-phosphate synthase (MIPS), have been demonstrated in the male reproductive organs of *D. melanogaster* and many mammals (Lewin *et al.* 1976; Loewus *et al.* 1983; Chauvin and Griswold 2004; Chintapalli *et al.* 2007). Inositol supplementation is known to reduce sterility, enhance early embryonic development, and reduce the incidence of neural tube defects in mammals (Ting *et al.* 2003; Alebous *et al.* 2009; Colazingari *et al.* 2014). Together this suggests that MIPS may play a role in reproduction and development.

### *Inos* gene deletions were constructed and confirmed

**Figure 1:**
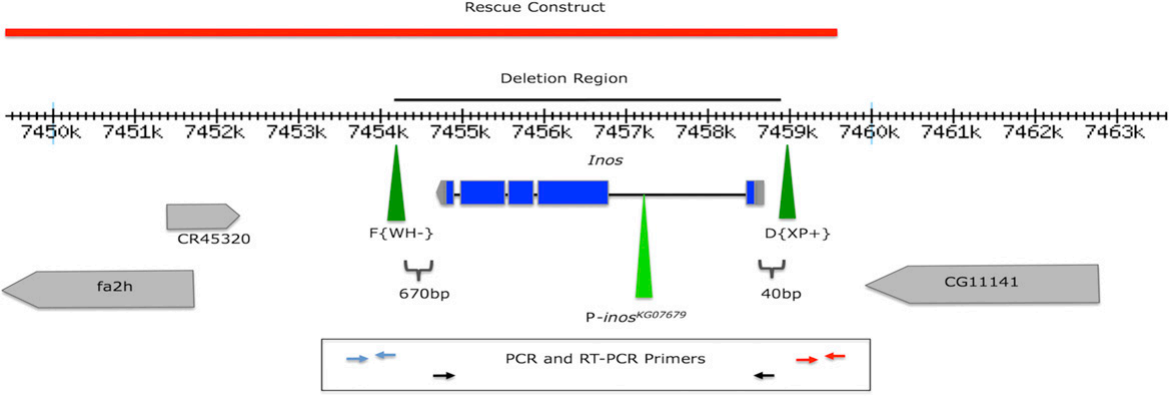
The *D. melanogaster Inos* gene and the surrounding genomic region of chromosome 2. Location of the FRT elements F{WH-} and D{XP+}, and P-*inos^KG07679^* (green arrowheads), rescue construct (CH322-61014) (red bar), the flanking genes (gray), WH- and downstream genomic DNA primers (blue arrows), XP+ and upstream genomic DNA primers (red arrows), and *Inos* 5′ UTR and *Inos* 3′ UTR primers (black arrows) are indicated. Exons are shown in blue, introns are black lines, UTRs are gray.

To directly test the involvement of MIPS in reproduction and development, the FLP-FRT system was used to generate a deletion of the *Inos* gene (which encodes MIPS) of *Drosophila melanogaster*. Initial evidence that recombination occurred, and that the *Inos* gene was deleted, was the appearance of dark red-eyed and white-eyed progeny when heat shocked hs-FLP males containing both the FRT-bearing insertion elements D{XP+} and F{WH-} were crossed to white-eyed (*w^1118^*) females. The insertion elements, initially *in trans* in males prior to recombination, are oriented so that recombination at the FRTs will generate chromosomes with two copies of the mini-white gene (deleting *Inos*) or no copies of the mini-white gene (with two copies of *Inos*). Three independently isolated putative deletion strains were recovered and outcrossed twice to white-eyed flies (*w^1118^/w^1118^*; *sna^Sco^*/CyO), and the dark red eyed curly-winged progeny were retained. PCR and sequencing results confirmed the generation of three identical *inos^ΔDF^* deletion strains. The 4,663 bp deletion extends from 40 bases upstream of the beginning of the 5′ UTR to 670 bases downstream of the end of the 3′ UTR (Figure 1).

### *Inos* deletion strains lack *Inos* transcript

**Figure 2:**
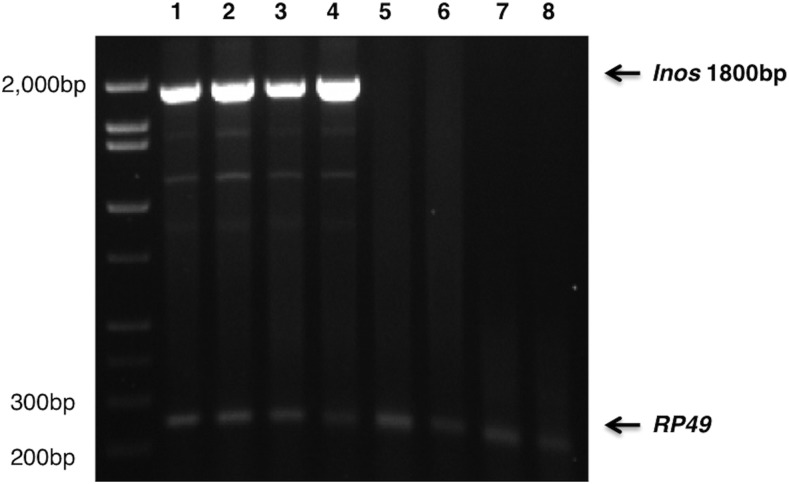
*Inos* deletion strains lack *Inos* transcript. RT- PCR analysis revealed one primary ∼1.8kb *Inos* transcript. RNA was extracted from (lane 1) wild-type females (OR), (lane 2) wild-type males (OR), (lane 3) *Inos* rescue strain females (*inos^ΔDF^*/ *inos^ΔDF^* ; res/res), (lane 4) *Inos* rescue strain males (*inos^ΔDF^*/ *inos^ΔDF^* ; res/res), (lane 5) homozygous P-*inos^KG07679^*/P-*inos^KG07679^* flies, (lane 6) biological replicate of lane 5, (lane 7) homozygous deletion flies (*inos^ΔDF^*/ *inos^ΔDF^*), (lane 8) biological replicate of lane 7.

RT-PCR was performed using primers designed to the 5′ and 3′ UTRs of the *Inos* transcript with poly(A)^+^ RNA from wild-type (OR), *Inos* rescue strain (homozygous deletion with an extra *Inos* gene on chromosome 3 (*inos^ΔDF^* / *inos^ΔDF^* ; res/res)), homozygous deletion (*inos^ΔDF^*/ *inos^ΔDF^*), or homozygous P-*inos^KG07679^*/P-*inos^KG07679^* flies (Figure 2). Primers to reverse transcribe and amplify *Rp49* (ribosomal protein L32) poly(A)^+^ RNA were used simultaneously to serve as internal experimental and loading controls. The wild-type flies express at least three different *Inos* transcripts, one major and multiple minor (Figure 2). All of the *Inos* transcripts evident in wild-type flies were absent from the homozygous deletion (*inos^ΔDF^*/ *inos^ΔDF^*) or homozygous P-*inos^KG07679^*/P-*inos^KG07679^* strains, and were present in the *Inos* rescue strain (*inos^ΔDF^*/ *inos^ΔDF^*; res/res) (Figure 2). One major and multiple minor *Inos* transcripts were observed when wild-type (CS or OR) cDNA was amplified with either MMLV-RT or AMV-RT. Although RNA (splice) isoforms detected with a single reverse transcriptase may be artifacts, Yu *et al.* demonstrated that transcripts detected with both MMLV-RT and AMV-RT prove to be consistently validated RNA isoforms (Yu *et al.* 2014). The biological role of these RNA isoforms remains to be explored. The dominant band (∼1.8 kb) was sequenced and aligned to the expected *Inos* transcript (99.9% identical). This transcript encodes the full-length MIPS protein expressed by Park and Kim (2004).

### *Inos* deletion affects development and longevity Without dietary inositol

**Figure 3:**
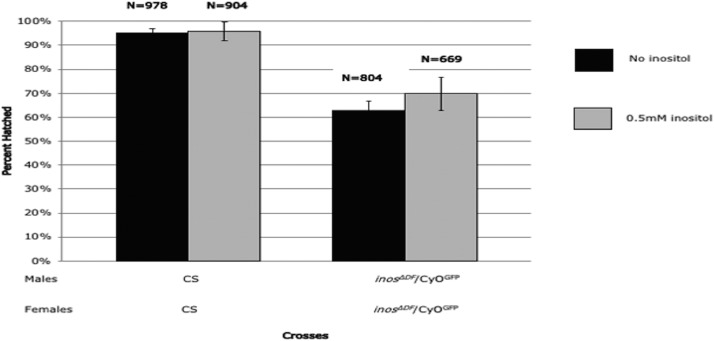
Lack of Dietary Inositol Does Not Affect Hatching Rate. The percentage of eggs hatched is indicated by the bars, and the number of eggs monitored (n) is indicated for each cross. Mean ± SE of five independent trials are represented. *P* > 0.6. As expected, since CyO^GFP^/CyO^GFP^ is lethal, the heterozygote hatching rate is approximately 75%.

**Figure 4:**
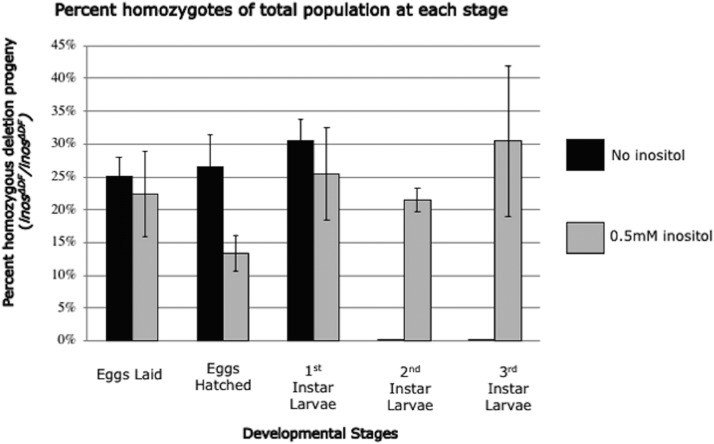
*inos^ΔDF^* deletion homozygotes die as first-instar larvae on chemically defined food without inositol. Percent of homozygotes (*inos^ΔDF^*/ *inos^ΔDF^*, GFP-negative) of the total population at each developmental stage. Mean ± SE of three independent trials are represented. *P* = 0.004.

To determine if inositol is essential for development, progeny of heterozygous deletion flies were grown with or without dietary inositol. Heterozygotes (*inos^ΔDF^*/ CyO^GFP^) with GFP-marked CyO balancers were distinguished from deletion homozygotes *(inos^ΔDF^*/ *inos^ΔDF^*, GFP-negative) throughout development. In these experiments, heterozygotes (*inos^ΔDF^*/ CyO^GFP^) were mated on chemically defined food. Absence of dietary inositol did not affect the proportion of eggs that hatched (*P* > 0.6, Figure 3). To determine if later development was affected, 257 eggs on chemically defined food with inositol and 349 eggs on chemically defined food without inositol were examined from deposition to adulthood. As expected, homozygous and heterozygous deletion progeny survived on the chemically defined food with inositol. The heterozygous deletion strain *inos^ΔDF^*/ CyO^GFP^ survived to adulthood without dietary inositol, as did the *Inos* rescue strain (*inos^ΔDF^*/ *inos^ΔDF^*; res/res). Figure 4 focuses on the homozygous deletion progeny (*inos^ΔDF^*/ *inos^ΔDF^*, GFP-negative), the proportion of which are displayed at each developmental stage. The remainder of the progeny (totaling 100% at each stage), GFP-containing heterozygotes *inos^ΔDF^*/ CyO^GFP^ and CyO^GFP^/CyO^GFP^ homozygotes, are not shown. Deletion homozygotes (*inos^ΔDF^*/ *inos^ΔDF^*) only survived to the first-instar larval stage on chemically defined food without dietary inositol. No homozygous *inos^ΔDF^*/ *inos^ΔDF^* deletion (GFP-negative) second-instar larvae were observed on chemically defined food without inositol (*P* = 0.004). Sufficient inositol or *Inos* transcript may have been transferred to the eggs allowing for survival of the deletion homozygotes through embryogenesis to the first-instar larval stage. Maternal transfer of *Inos* transcript was ruled out by other experiments in which homozygous *inos^ΔDF^*/ *inos^ΔDF^* deletion females were mated to heterozygous *inos^ΔDF^*/ CyO^GFP^ males on rich food supplemented with inositol. All the progeny survived on this rich food supplemented with inositol, demonstrating that *Inos* transcript is not essential for development. So maternally supplied inositol, without *Inos* transcript, is sufficient to support development to the first larval-instar stage. Although the precise cause of death during the first-instar stage is not known, inositol is likely to be needed for membrane biogenesis and/or signal transduction.

**Figure 5:**
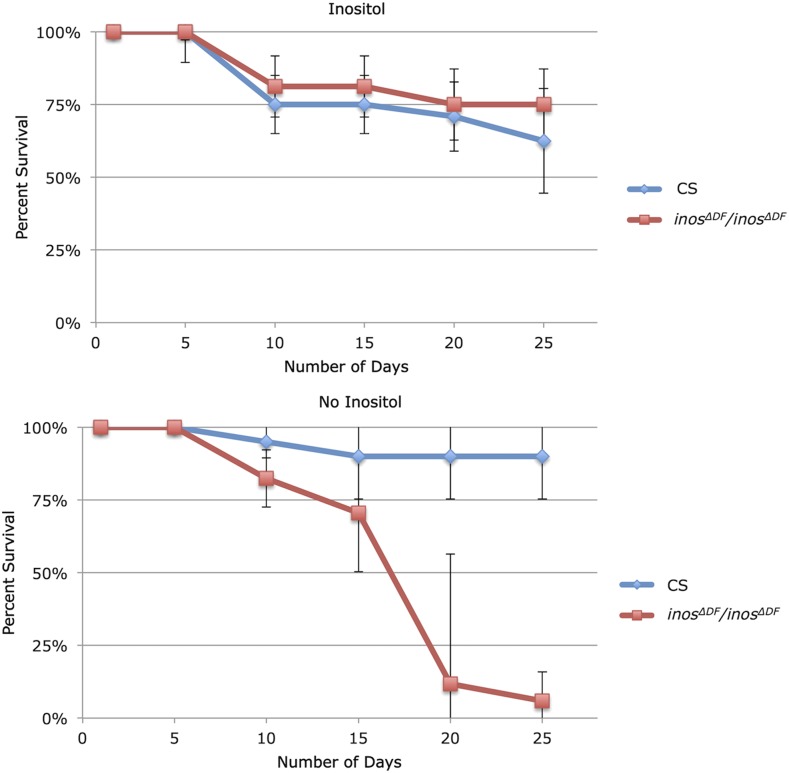
Homozygous *inos^ΔDF^* deletion flies die without dietary inositol on chemically defined food. Homozygous *inos^ΔDF^* deletion flies and wild-type (CS) flies grown on chemically defined food with 0.5mM inositol (top panel, *P* = 0.302) or without inositol (bottom panel, *P* = 8.23 × 10^−16^). Mean ± SE of three independent trials are represented.

The longevity of homozygous *inos^ΔDF^* deletion flies was compared to wild-type (CS) flies. Pupae were transferred to chemically defined food with and without inositol and survival was monitored. A total of approximately two hundred adults were examined, at least forty for each strain on each food. As expected, the life span of homozygous *inos^ΔDF^* deletion flies was significantly greater on chemically defined food with inositol than without inositol (Figure 5). Even without inositol most of the homozygous *inos^ΔDF^* deletion flies survived nearly a week. This was not surprising, as the inositol accumulated by larvae grown on rich food could support survival but would eventually be depleted (Aguila *et al.* 2013). Equal numbers of males and females were examined and no differences were observed. The *p*-value of 8.23 × 10^−16^ indicates that the difference in life span between homozygous *inos^ΔDF^* deletion and wild-type (CS) flies on chemically defined food, without inositol, is statistically significant. The rescue *Inos* construct restored wild-type like development, growth, and survival to homozygous *inos^ΔDF^* deletion flies grown on chemically defined food without inositol. There is no significant difference between survival of homozygous *inos^ΔDF^* deletion and wild-type (CS) flies on chemically defined food supplemented with inositol (*P* = 0.302, Figure 5).

### Deletion flies fail to establish a homozygous stock even With dietary inositol

**Figure 6:**
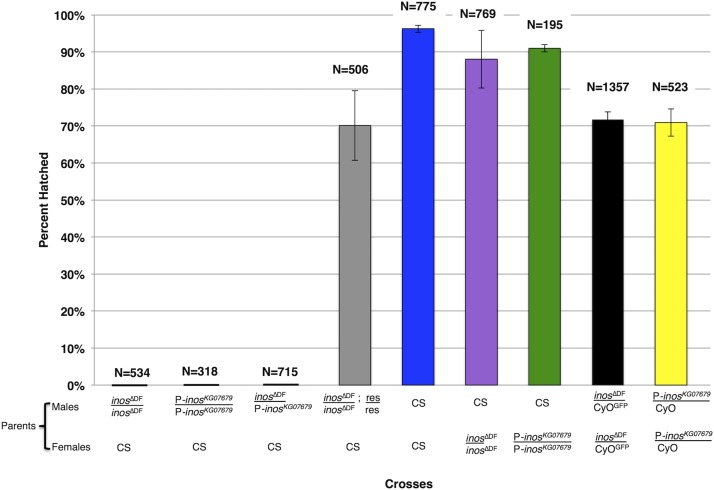
Deletion of *Inos* causes male sterility. Strains indicated were grown and mated on rich food supplemented with 170mM inositol. The percentage of eggs hatched is indicated by the bars, and the number of eggs monitored (n) is indicated for each cross. Mean ± SE of three independent trials are represented.

Although rich food with inositol restored viability, the homozygous *inos^ΔDF^* deletion flies failed to establish a homozygous stock. To determine whether the males, females, or both were sterile, homozygous *inos^ΔDF^* deletion, heterozygotes, and wild-type (CS) flies were mated on rich food supplemented with 170mM inositol. Deletion homozygous females mated to wild-type (CS) males laid eggs that hatched normally. The number of eggs laid and the number of eggs hatched 48 hr later were recorded (Figure 6). When homozygous deletion (*inos^ΔDF^*/ *inos^ΔDF^*) males were mated to wild-type (CS) females, none of the eggs hatched suggesting that these males were sterile. The *Inos* rescue construct restored fertility to these males (*inos^ΔDF^*/ *inos^ΔDF^*; res/res) with 71% of the eggs hatching. This is significantly different than the results obtained with wild-type (CS) males (*P* = 1.69 × 10^−10^). Perhaps not all the upstream promoter/enhancer elements are included in the rescue construct. Position effects of the extra *Inos* construct may also be responsible for the less than 100% rescue of male fertility (McElroy *et al.* 2014). When homozygous *inos^ΔDF^* deletion females were mated to wild-type (CS) males, 88% of the eggs hatched. As expected, approximately 75% of the eggs hatched when heterozygous deletion (*inos^ΔDF^*/CyO^GFP^) females were mated with heterozygous deletion (*inos^ΔDF^*/CyO^GFP^) males, as CyO^GFP^/CyO^GFP^ is lethal.

### *P*-element insertion Into the *Inos* gene also causes male sterility even With dietary inositol

The phenotype of a strain containing a *P*-element in the first intron of the *Inos* gene was compared to the *inos^ΔDF^* deletion strain. Similar to the homozygous *inos^ΔDF^* deletion flies, the life span of homozygous *P*-element (P-*inos^KG07679^*/P-*inos^KG07679^*) deletion flies was significantly greater on chemically defined food with inositol than without inositol (data not shown). Moreover, both the homozygous *inos^ΔDF^* deletion males and the homozygous P-*inos^KG07679^* males were sterile. None of the eggs hatched when homozygous P-*inos^KG07679^* were mated to wild-type (CS) females on rich food supplemented with 170mM inositol (Figure 6). In contrast, when homozygous P-*inos^KG07679^* females were mated to wild-type (CS) males 92% of the eggs hatched (Figure 6). This is consistent with data published in 1993 describing a *P*-element insertion located in band 43C near the *Inos* gene region, which also caused male sterility (Castrillon *et al.* 1993). Unfortunately, the specific location of the *P*-element in the publication was not determined, so in 1993 the male sterile phenotype was not directly attributed to the disruption of the *Inos* gene. To demonstrate that the male sterile phenotype is due to disruption of *Inos* gene function, P-*inos^KG07679^*/ *inos^ΔDF^* were crossed to wild-type (CS) flies. When P-*inos^KG07679^*/ *inos^ΔDF^* males were mated to wild-type (CS) females, none of the eggs hatched. This demonstrates that the P-*inos^KG07679^* fails to complement the sterility of the *inos^ΔDF^* deletion (Figure 6). When heterozygous P-*inos^KG07679^*/CyO females are mated to heterozygous P-*inos^KG07679^*/CyO males, more than 70% of the eggs hatch, consistent with the expected results since CyO/CyO is lethal.

### Wild-type females mated to deletion males have virgin-Like ovaries and seminal receptacles

**Figure 7:**
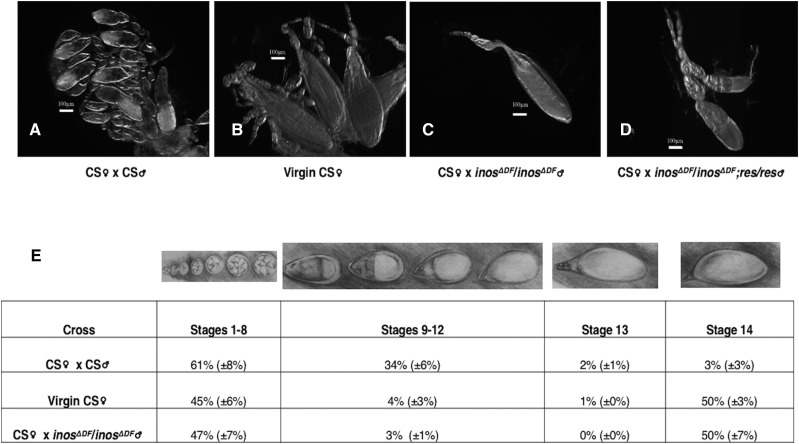
Ovarioles of females mated to deletion males look virgin-like. Dissected ovarioles from wild-type (CS) females imaged with differential interference contrast microscopy. A) mated to wild-type (CS) males, B) virgin, C) mated to homozygous deletion males (*inos^ΔDF^* / *inos^ΔDF^*), D) mated to *Inos* rescue strain (*inos^ΔDF^* / *inos^ΔDF^*;res/res) males, and E) drawn images of oogenesis stages and table displaying percentage of each stage present in entire ovaries. The number of ovarioles per ovary is similar among the strains, fewer are shown in this figure for image clarity. Mean ± SE of three independent trials are represented. Scale bars correspond to 100 μm.

After growth on rich food, ovaries dissected from wild-type (CS) females mated to homozygous *inos^ΔDF^* deletion males exhibited a phenotype similar to virgin wild-type (CS) females (Figure 7). In virgin wild-type females, mature stage 14 oocytes accumulate, oocyte development becomes blocked, and stages 9 through 13 of oogenesis seem to disappear (Spradling 1993). To quantify the proportion of each stage, entire ovaries were examined and the number of egg chambers was tabulated (Figure 7E). The proportion of stages 9 through 13 was greatly reduced in the ovarioles of wild-type (CS) females mated to the homozygous *inos^ΔDF^* deletion males, similar to wild-type virgin females. In the reciprocal experiments, when homozygous *inos^ΔDF^* deletion females, also grown with dietary inositol, were mated with wild-type (CS) males, normal ovary morphology was observed. These data suggest that mating to homozygous deletion males does not release wild-type females from the previtellogenic block observed in virgins that have accumulated mature oocytes. This is particularly interesting since other studies have shown that mating to males with sterility caused by mutations in some other genes is known to overcome this block and, although no progeny are produced, the females no longer have virgin-like ovaries (Spradling 1993).

Homozygous *inos^ΔDF^* deletion males were observed to exhibit normal mating behavior including copulation. Since the eggs laid by wild-type (CS) females mated to these males failed to hatch (Figure 6), they were examined by confocal microscopy after staining with DAPI. These eggs did not appear to be fertilized (data not shown) so the transfer of sperm was examined next. No sperm were apparent in the seminal receptacle of wild-type (CS) females after copulation with homozygous *inos^ΔDF^* deletion males, yet sperm were readily apparent when mated to wild-type (CS) males. In addition, deletion of *Inos* may alter some of the seminal fluid components (Heifetz *et al.* 2001; Chow *et al.* 2015). In many organisms the unfolded protein response pathway, which alleviates endoplasmic reticulum (ER) stress, is known to be activated by inositol deprivation. The sterility associated with ER stressed *D. melanogaster* males, however, is different than the phenotype described here. The ER stressed males have normal sperm production and transfer (Chow *et al.* 2015) yet (as described above) the homozygous *inos^ΔDF^* deletion males in this study do not have normal sperm transfer with no sperm evident in the seminal receptacles of their mating partners.

### Testes From deletion flies lack sperm in the seminal vesicles

**Figure 8:**
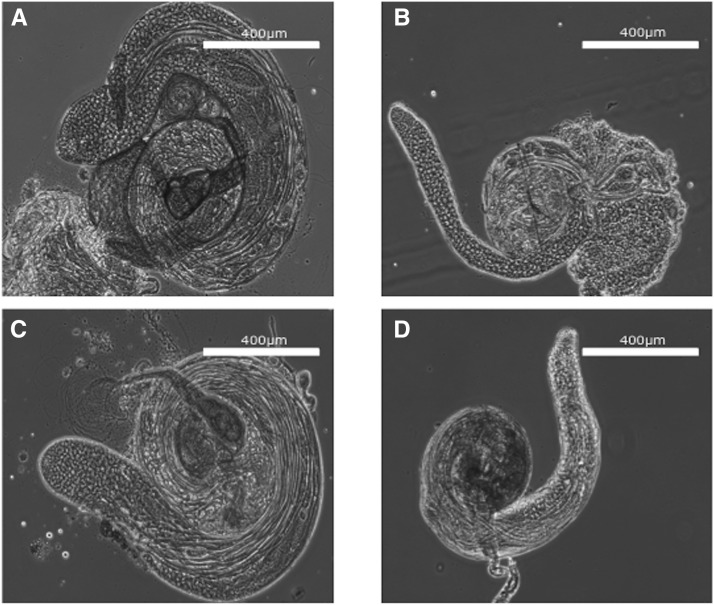
Testes of homozygous *inos^ΔDF^* deletion males are smaller than wild-type males’ testes. Phase contrast microscopy (representative images) of testes dissected from 2-day-old (A) wild-type (CS), (B) homozygous deletion (*inos^ΔDF^* / *inos^ΔDF^*), (C) *Inos* rescue strain (*inos^ΔDF^* / *inos^ΔDF^*;res/res), and (D) homozygous P-*inos^KG07679^*/P-*inos^KG07679^* male flies. Scale bars correspond to 400 μm.

**Figure 9:**
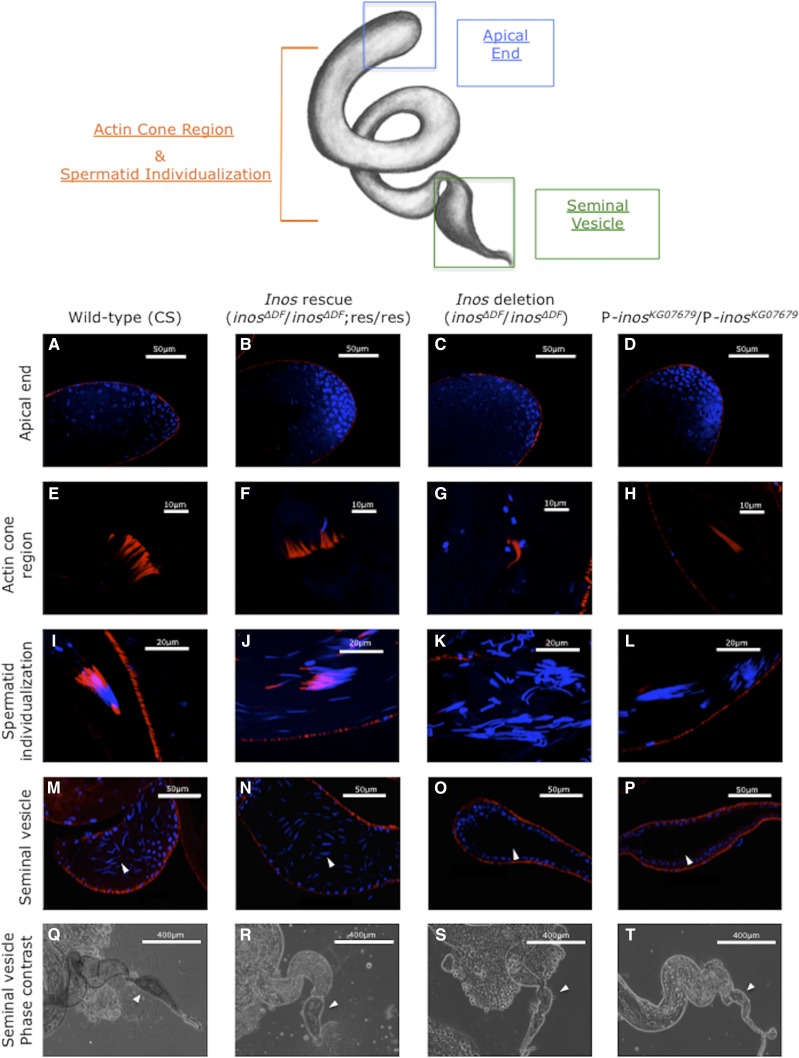
Homozygous *inos^ΔDF^* deletion males lack sperm in the seminal vesicles. Microscopy of testes dissected from 2-day-old male flies. Rows 1-4, confocal imaging of samples stained with DAPI (blue) and rhodamine phalloidin (red). Top row (apical end) scale bars are 50 μm (A-D), second row (actin cone region) scale bars are 10 μm (E-H), third row (spermatid individualization) scale bars are 20 μm (I-L), and fourth row (seminal vesicle, arrowheads) scale bars are 50 μm (M-P). Row 5 contains phase contrast images (base of testis and seminal vesicle, arrowheads), the scale bars are 400 μm (Q-T).

To determine whether spermatogenesis was disrupted, testes of wild-type (CS), homozygous deletion (*inos^ΔDF^*/ *inos^ΔDF^*), homozygous deletion with an extra *Inos* construct on chromosome 3 (*inos^ΔDF^*/ *inos^ΔDF^*; res/res), and homozygous P-*inos ^KG07679^*/P-*inos^KG07679^* males were dissected. Testes of homozygous deletion (*inos^ΔDF^*/ *inos^ΔDF^*) and homozygous P-*inos ^KG07679^*/P-*inos^KG07679^* males were overall smaller and more fragile (Figure 8). No mature sperm were detected in the seminal vesicles of homozygous *inos^ΔDF^*/ *inos^ΔDF^* or homozygous P-*inos^KG07679^*/P-*inos^KG07679^* males (Figure 9O, P, S, T). This phenotype is completely penetrant. Both homozygous deletion flies (*inos^ΔDF^*/ *inos^ΔDF^*) and homozygous P-*inos ^KG07679^*/P-*inos^KG07679^* flies have irregularly shaped spermatid bundles (Figure 9K, L), indicating a disruption of individualization. In both homozygous mutant stains, the first visible defect is in the alignment of the nuclei and the failure of the actin cones to form (Figure 9G, H). The actin cones are extremely rarely visible, and never seen in a bundle. Three of eleven homozygous *inos^ΔDF^* deletion males show only a single cone in one of the testes, the rest show no actin cones. In contrast, in every wild-type (CS) control testis many bundles of approximately 30-64 actin cones are evident in the plane of the confocal images. Actin cones are usually found in tight parallel bundles, which promote the maturation of spermatids. During this process, actin cones remove excess cytoplasm and organelles while enclosing each spermatid in its own membrane (Couderc *et al.* 2017, Isaji *et al.* 2011, Steinhauer 2015). Disruption of *myo*-inositol synthesis results in nearly no actin cone formation. Consistent with the lack of actin cones, no waste bags were ever observed in testes of either homozygous mutant strain. The testes of homozygous deletion males with the *Inos* rescue construct on chromosome 3 (*inos^ΔDF^*/ *inos^ΔDF^*; res/res) (Figure 9B, F, J, N, R) appear similar to wild-type (CS) (Figure 9A, E, I, M, Q), with sperm evident in the seminal vesicles.

The lack of sperm in the seminal vesicles in homozygous *inos^ΔDF^* deletion males is consistent with failure at individualization. Many male sterile mutants of *Drosophila melanogaster* exhibit a “classical” phenotype of abnormalities at individualization, which reflects the arrest of spermatogenesis at a checkpoint (Wakimoto *et al.* 2004). So, the defect due to lack of inositol might be at an earlier stage of spermatogenesis.

In *D. melanogaster* spermatogenesis, primary spermatogonial cells begin their descent from the tip of the testis and are enclosed in a pair of cyst cells that serve as somatic support cells and restrictive permeability barriers. Testicular barriers are highly selective to prevent pathogens or possible mutagens from harming developing sperm. The soma-germline barrier provided by this cyst cell encapsulation becomes increasingly stringent as the sperm progress through development (Fairchild *et al.* 2015). It appears that while encapsulated, the developing spermatogonial cells cannot use nutritionally acquired *myo*-inositol, forcing dependence on MIPS. Without inositol synthesis, and unable to access ingested inositol due to a testicular-like barrier, the homozygous deletion flies had no sperm in their seminal vesicles and were sterile.

## Broader Implications

In humans, two common causes of infertility are oligoasthenoteratozoospermia and polycystic ovary syndrome (PCOS). The addition of *myo*-inositol to oligoasthenoteratozoospermia sperm has been reported to correct their morphological abnormalities and to increase their ability to fertilize eggs *in vitro* (Bevilacqua *et al.* 2015; Simi *et al.* 2017). Follicular fluid from women with PCOS has been shown to have a significantly reduced level of *myo*-inositol. Supplementation of these women with *myo*-inositol enhances their egg quality and fertility (Unfer *et al.* 2011; Colazingari *et al.* 2013; Nas and Tuu 2017). Clearly these human fertility conditions are related to inositol metabolism. This study demonstrates the requirement for inositol in reproduction in *Drosophila melanogaster*, and validates this organism as a model for the further examination of the role *myo*-inositol synthesis in reproduction.
